# Improved Functional Organization in Patients With Primary Insomnia After Individually-Targeted Transcranial Magnetic Stimulation

**DOI:** 10.3389/fnins.2022.859440

**Published:** 2022-03-10

**Authors:** Shun Qi, Yao Zhang, Xiang Li, Chuanzhu Sun, Xiaowei Ma, Sanzhong Li, Li Li, Kai Ren, Min Xi, Zi-Gang Huang

**Affiliations:** ^1^The Key Laboratory of Biomedical Information Engineering of Ministry of Education, Institute of Health and Rehabilitation Science, School of Life Science and Technology, Research Center for Brain-Inspired Intelligence, Xi’an Jiaotong University, Xi’an, China; ^2^Shaanxi Brain Modulation and Scientific Research Center, Xi’an, China; ^3^Xijing Hospital, The Air Force Military Medical University, Xi’an, China; ^4^The Key Laboratory of Biomedical Information Engineering of Ministry of Education, Institute of Health and Rehabilitation Science, School of Life Sciences and Technology, Xi’an Jiaotong University, Xi’an, China; ^5^The Key Laboratory of Neuro-Informatics and Rehabilitation Engineering of Ministry of Civil Affairs, Xi’an, China; ^6^Xi’an Solide Brain Control Medical Technology Company, Xi’an, China; ^7^Department of Nuclear Medicine, The Second Xiangya Hospital, Central South University, Changsha, China; ^8^Department of Neurosurgery, Xijing Hospital, The Air Force Military Medical University, Xi’an, China; ^9^Center of Treatment and Rehabilitation of Severe Neurological Disorders, Xi’an International Medical Center Hospital, Xi’an, China; ^10^Department of Cardiovascular Surgery, Xijing Hospital, Air Force Military Medical University, Xi’an, China; ^11^Hospital of Northwestern Polytechnical University, Xi’an, China; ^12^The State Key Laboratory of Cognitive Neuroscience and Learning, Beijing Normal University, Beijing, China

**Keywords:** primary insomnia, repetitive transcranial magnetic stimulation, small-world property, treatment effects, fMRI

## Abstract

Primary insomnia (PI) is among the most prevalent sleep-related disorders and has a far-reaching impact on daytime functioning. Repetitive transcranial magnetic stimulation (rTMS) has drawn attention because of its effectiveness and safety. The purpose of the current study was to detect changes in the topological organization of whole-brain functional networks and to determine their associations with the clinical treatment effects of rTMS. Resting-state functional magnetic resonance imaging (rsfMRI) data from 32 patients with PI were collected and compared with findings from 32 age- and gender-matched healthy controls (HCs). The patients were treated with Stanford accelerated intelligent neuromodulation therapy, which is a recently validated neuroscience-informed accelerated intermittent theta-burst stimulation protocol. Graph theoretical analysis was used to construct functional connectivity matrices and to extract the attribute features of small-world networks in insomnia. Scores on the Insomnia Severity Index (ISI), Pittsburgh Sleep Quality Index, Self-Rating Anxiety Scale, Self-Rating Depression Scale, and the associations between these clinical characteristics and functional metrics, were the primary outcomes. At baseline, the patients with PI showed inefficient small-world property and aberrant functional segregation and functional integration compared with the HCs. These properties showed renormalization after individualized rTMS treatment. Furthermore, low functional connectivity between the right insula and left medial frontal gyrus correlated with improvement in ISI scores. We highlight functional network dysfunctions in PI patients and provide evidence into the pathophysiological mechanisms involved and the possible mode of action of rTMS.

## Introduction

Primary insomnia (PI) is an important public health problem worldwide, with nearly one-third of the general population experiencing insomnia symptoms during their lifetime (Xue et al., 2022). PI is characterized by difficulty falling asleep, reduced sleep depth, and early awakening. Lack of sleep can lead to various health problems such as cardiovascular disease (Palagini et al., 2013), obesity (Hargens et al., 2013), diabetes, immune system dysfunction, and cognitive and affective disorders. Cognitive behavioral therapy (CBT) and medication are the most commonly used treatments for PI, although they both have some shortcomings. CBT requires a long treatment duration, has a high cost, and shows limited efficacy (Okajima et al., 2017), whereas drugs may bring about adverse effects such as memory disorder, delirium, and daytime sleepiness (Hrehová and Mezian, 2021). As such, there is an urgent need for new therapies for insomnia that have minimal side effects and can be used over a long term.

If delivered repetitively, transcranial magnetic stimulation (TMS) can transiently modulate the excitability and plasticity of stimulated areas and other areas connected to them. Repetitive TMS (rTMS) has been used with varying degrees of effectiveness in many neurological and psychiatric diseases, and is emerging as a promising tool for treating several sleep disorders (Nardone et al., 2020). Indeed, rTMS can improve sleep quality, optimize sleep structure, and maintain therapeutic efficacy to a greater extent than pharmacological treatments and cognitive behavioral interventions (Babiloni et al., 2021). The effects of rTMS depend on the intensity, frequency, and number of pulses delivered, the duration of the course, the coil location, and the type of coil employed. The left dorsolateral prefrontal cortex (DLPFC) is the key TMS targeting area for treating PI. The first rTMS study in PI found that the relapse and recurrence rates within 3 months were the lowest in rTMS treatment group compared with the medication treatment group and psychotherapy treatment group (Jiang et al., 2013). A recent study indicated that rTMS combined with acupuncture can effectively enhance sleep quality in chronic PI patients (Zhang et al., 2018). However, conventional TMS treatments for PI lack personalized intervention. If the DLPFC can be stimulated accurately every time and quantization is consistent, rTMS may have a good effect. Recently, the Stanford Accelerated Intelligent Neuromodulation Therapy (SAINT), which is an accelerated fMRI–guided intermittent theta-burst stimulation (iTBS) protocol, was shown to be an effective, safe, tolerable, and rapid acting therapy for treatment-resistant depression (Cole et al., 2020, 2021). Insomnia is a risk factor for depression, the treatment of which offers the critical opportunity to prevent major depressive episodes. Previous studies have indicated that depression and insomnia may share a common genetic background and biologic mechanisms that involve abnormal DLPFC activities (Plante, 2021). Stimulation of DLPFC that is most anticorrelated with the subgenual anterior cingulate cortex (sgACC) in each individual has recently been shown to alleviate treatment-resistant depression (Cole et al., 2020, 2021), furthermore, impaired activities within sgACC were also commonly reported in PI patients (Yan et al., 2018; Zhu et al., 2021), Whether SAINT protocol will also show promising treatment effects for PI needs to be determined.

Previous studies found that amygdala reactivity, morphometry, and adaptation are altered in insomnia, indicating that the processing of negative stimuli is intensified and longer lasting (Huang et al., 2012). Additionally, patients with insomnia show aberrant connectivity in the default mode network (DMN) (Pang et al., 2017) and salience network (SN) (Chen et al., 2014), which is associated with subjective sleep disturbances, hyperarousal, maladaptive emotion regulation, and disturbed integration of emotional states. The limbic circuit is assumed to play a crucial role in enhanced recall of negative experiences, and activation likelihood estimation (ALE) analysis indicated that PI patients showed significant gray matter reduction in the right middle frontal gyrus (Tahmasian et al., 2018). These results indicate that abnormality in the widespread functional network might contribute to the pathogenesis of PI.

In this study, we applied SAINT therapy with “individualized stimulation” and personalized DLPFC location as an intervention for patients with PI. Because PI is considered to be a functional disorder with abnormal brain network connections, we hypothesized that functional network efficiency will show improved connectivity after application of the SAINT protocol.

## Materials and Methods

### Subjects

Patients with PI were recruited from the Xijing Hospital. All patients met the diagnostic criteria for insomnia in the DSM-IV diagnostic criteria. Age- and gender-matched healthy control subjects were recruited from the local community. The exclusion criteria common to both groups were: (1) mental diseases; (2) shift workers; (3) other sleep disorders, such as sleep apnea; (4) body mass index (BMI) ≥ 30; (5) history or presence of significant neurological or medical illnesses; (6) history of alcohol, drug, or smoking abuse; (7) contraindications for 3-T MRI, such as claustrophobia, metal implants, and pacemakers; and (8) female patients who were pregnant, nursing, or menstruating. The current study was approved by the Research Ethics Review Board of the Institute of Mental Health of the Xijing Hospital. Informed written consent was obtained from each subject enrolled in the study.

### Magnetic Resonance Imaging Data Collection

A 3.0-T UNITED Discovery 770 MRI scanner was used for all MRI acquisitions. Participants were required to keep still and stay awake during the entire session. All scans were performed between 18:00 and 20:00. The resting-state functional images were obtained with the following parameters: field of view (FOV) = 224 mm × 224 mm, data matrix = 64 × 64, echo time (TE) = 30 ms, repetition time (TR) = 2,000 ms, slice thickness = 4 mm, flip angle = 90° and voxel size = 3.5 mm^3^ × 3.5 mm^3^ × 40 mm^3^. For anatomical reference, a high-resolution T1-weighted image was also acquired with the following parameters: TR = 7.24 ms, TE = 3.10 ms, FOV = 256 mm × 256 mm, flip angle = 10°, slice thickness = 0.5 mm, and voxel size = 0.5 mm × 0.5 mm × 1 mm. The same parameters were used for follow-up scans of the PI patients and healthy controls.

### Functional Magnetic Resonance Imaging Data Preprocessing

The resting-state fMRI images were preprocessed using Data Processing and Analysis for Brain imaging (DPABI)^1^ software, which reproduces procedures in the Resting State fMRI Data Analysis Toolkit (REST)^2^ and Statistical Parametric Mapping (SPM12).^3^ The first 10 images were removed to allow for magnetization equilibrium, then the remaining 200 images were subjected to slice timing correction and motion realignment, during which the mean frame-wise displacement (FD) was calculated. Subjects with maximal translation of more than 2 mm or maximal rotation of more than 2° were excluded. Then, the Friston-24 model was used to regress out the nuisance signals from cerebrospinal fluid, white matter head motion effects. The fMRI data were then normalized into MNI (Montreal Neurological Institute) space using the DARTEL (diffeomorphic anatomical registration through exponentiated lie algebra) method, smoothed with a Gaussian kernel of 6 mm full-width-at-half-maximum, and band-pass filtered (0.01–0.08 Hz).

### Treatment

Repetitive transcranial magnetic stimulation was delivered using a commercially available magnetic stimulator (Black Dolphin Navigation Robot). Individual DLPFC stimulation targets were determined using a method similar to that in a previous study (Cole et al., 2021). First, a hierarchical agglomerative clustering algorithm was used to divide the DLPFC and sgACC into numerous functional subunits defined according to correlated voxel pairs. For each functional subunit, a single time-series value representing the time-series that correlated most strongly with the median time series was identified. Then, a matrix of inter-subunit Spearman correlation coefficients was calculated. Finally, the optimal target in the DLPFC was determined by considering the anticorrelation, size, spatial concentration, and dispersion of subunits. Fifty intermittent theta-burst stimulation (iTBS) sessions (1,800 pulses per session, 50-min interval) were delivered as 10 daily sessions over five consecutive days at 90% resting motor threshold.

### Network Construction

GRETNA software^4^ was used to analyze brain network properties according to graph theory. Using the automated anatomical labeling (AAL) atlas, the whole brain was divided into 116 network nodes. Pearson correlation coefficients between the time series of all possible pairs of nodes were calculated, yielding a 116 × 116 correlation matrix for each participant. Similar with a previous study (Song et al., 2020), the correlation matrix of each subject was then transformed into an undirected binarized matrix using sparsity thresholding (8% ≤ s ≤ 50%) at intervals of 0.01. This was to ensure that the number of nodes and connections were matched across participants. The functional segregation metrics of clustering coefficient (Cp), normalized clustering coefficient (γ), and local efficiency (Eloc), the functional integration metrics of characteristic path length (Lp), normalized characteristic path length (λ), and global efficiency (Eglob), and the small-worldness metric of σ (σ = γ/λ), were then obtained. The area under the curve (AUC) for each network metric was calculated for further statistical comparisons.

### Statistical Analysis

Differences in demographic characteristics between patients with PI and HCs were compared using the chi-square test and Student’s *t*-test performed with SPSS (IBM SPSS Statistics for Windows, version 18.0, IBM Corp.). Two-sample *t-*tests (HCs vs PPD patients at baseline; HCs vs PPD patients at follow-up) or paired *t*-tests (baseline vs follow-up) were used to identify changes in network metrics. The threshold for significance was set at *P* < 0.05, corrected for multiple comparisons using the false discovery rate (FDR) criterion. Age, gender, and mean FD calculated during the preprocessing steps were accounted for by including them as covariates. Finally, a network-based statistic (NBS) approach was used to determine any significant differences in functional connectivity across the three groups of PI at baseline, HCs, and PI at follow-up. The AUC values of each network metric showing abnormal differences (baseline vs follow-up) were extracted, then Pearson correlation (performed using SPSS) was used to examine associations between changes in network metrics and clinical scores. Correction for multiple comparisons was accomplished with the FDR criterion.

## Results

### Demographic Information

All participants (patients with PI and healthy controls) were right-handed. There was no significant difference in age, gender, and year of education between PI patients and HCs. As expected, the patients with PI exhibited significantly higher PSQI scores (*P* < 0.001), ISI scores (*P* < 0.001) self-rating of anxiety scores (*P* < 0.001), and self-rating of depression scores (*P* < 0.001) than the HCs. After rTMS treatment, all scores showed a significant improvement. Detailed information is listed in Table 1. The head motion indicated by mean FD did not differ significantly between baseline and follow-up in patients with PI (*p* > 0.05; mean FD = 0.137 ± 0.031 for baseline, mean FD = 0.129 ± 0.025 for follow-up), or between patients with PI and HCs (all *p* > 0.05; mean FD = 0.128 ± 0.019 for HCs).

**TABLE 1:** Demographic and clinical characteristics of participants.

Characteristics	PI (32)	HCs (32)	*p*
Age (years)	39.5 ± 6.4	39.7 ± 6.3	0.81
Gender (M/F)	17/15	16/16	0.80^+^
Education (years)	14.7 ± 3.5	15.1 ± 2.4	0.55
Duration of illness (months)	23.2 ± 15.3	–	–
PSQI	12.7 ± 3.4	5.75 ± 1.3	<*0*.*01*
ISI	20.6 ± 3.3	4.25 ± 2.3	<*0*.*01*
SAS	51.1 ± 8.4	40.6 ± 7.3	<*0*.*01*
SDS	55.8 ± 9.2	42.1 ± 6.8	<*0*.*01*
Characteristics	PI at baseline	PI at follow-up	
PSQI	12.7 ± 3.4	6.8 ± 2.2	<*0*.*01*
ISI	20.6 ± 3.3	7.0 ± 2.9	<*0*.*01*
SAS	51.1 ± 8.4	45.6 ± 6.5	<*0*.*01*
SDS	55.8 ± 9.2	46.1 ± 7.1	<*0*.*01*

*PI, primary insomnia; HCs, healthy controls; PSQI, Pittsburgh Sleep Quality Index; ISI, Insomnia Severity Index; SAS, Self-Rating Anxiety Scale; SDS, Self-Rating Depression Scale.*

*^+^The p-value was carried using Chi-square test.*

### Small-World Properties

**FIGURE 1:**
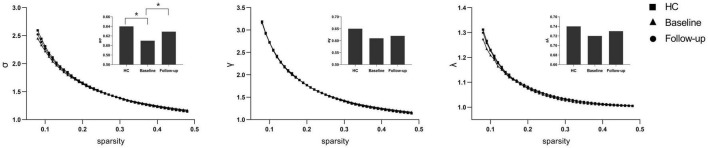
The typical small-world network architectures (γ > 1, λ ≈ 1, and σ > 1) across the sparsity. The square stands for the healthy controls (HC), the triangle stands for the PI patients at baseline and the circular stands for primary insomnia (PI) children at follow-up. Significant differences were found for the σ metric between HC and PI patients at baseline (*P* < 0.01) and between PI patients at baseline and at follow-up (*P < 0.01).

**FIGURE 2:**
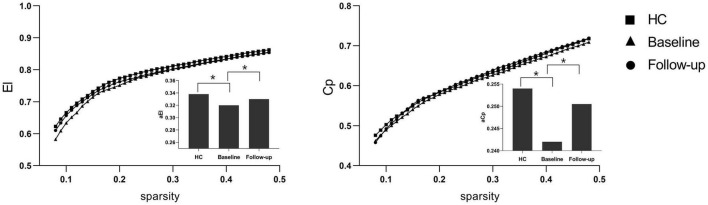
Functional segregation metrics Cp and El. The bar graph shows the value of significant AUC of the functional segregation parameters among the three groups. Black asterisks indicate significant differences (*P* < 0.01).

**FIGURE 3:**
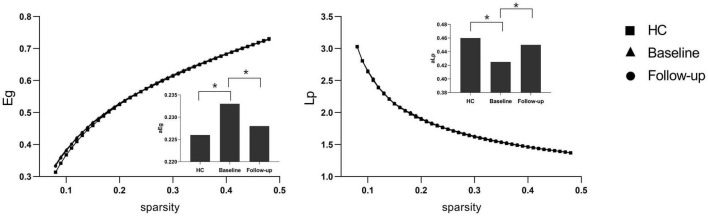
Functional integration metrics Lp and Eg. The bar graph shows the value of significant area under the curve (AUC) of the functional segregation parameters among the three groups. Black asterisks indicate significant differences (*P* < 0.01).

Under a sparsity of 0.08–0.48, the functional networks of all three groups (PI at baseline, HCs, and PI at follow-up) were characterized by small-world properties (σ > 1, λ > 1, γ > 1). Two-sample *t*-tests indicated that PI patients at baseline showed reduced small-world properties (σ, Figure 1), reduced functional segregation metrics (Cp and El, Figure 2), and reduced functional integration metrics (decreased Lp and increased Eg, Figure 3) compared with the HCs, whereas paired *t*-tests indicated that follow-up values (in the patients with PI) for these metrics showed an increase on baseline values, but no significant differences between HCs.

### Connectivity and Correlation Results

**FIGURE 4:**
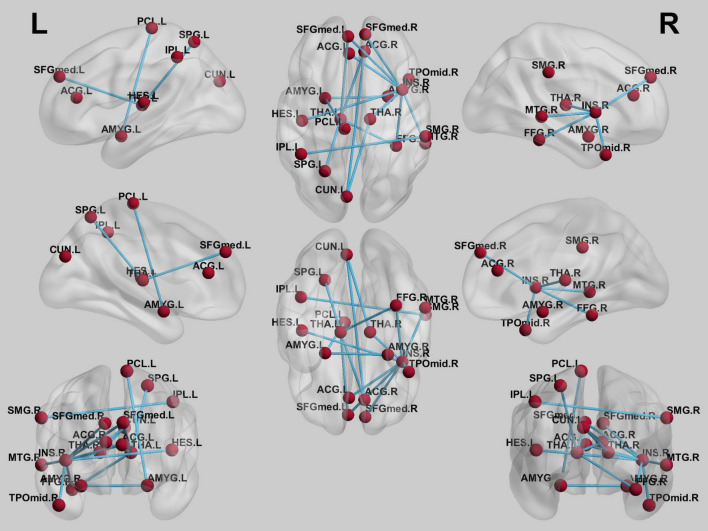
Reduced connectivity after repetitive transcranial magnetic stimulation (rTMS) treatment.

**FIGURE 5:**
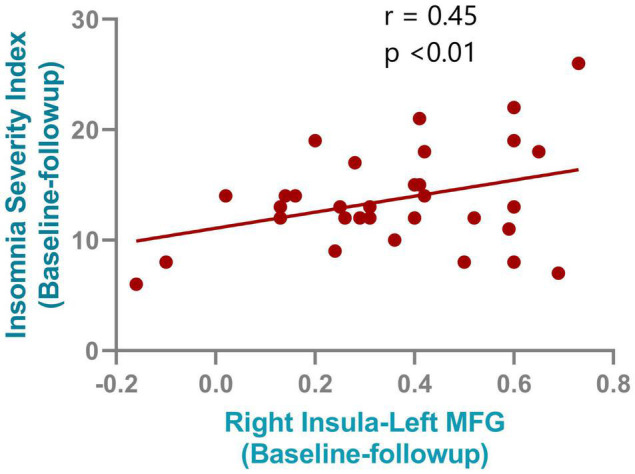
Correlation results between the connectivity change from right insula and left medial frontal gyrus and the insomnia severity index improvements after rTMS treatment.

After NBS multiple correction (*P* < 0.05), the PI patients showed reduced functional connections at follow-up compared with baseline values. The main nodes were located at the anterior cingulate gyrus (ACG), medial superior frontal gyrus, superior and inferior parietal gyrus, middle temporal gyrus, thalamus, and insula. Detailed connections (edges) are shown in Figure 4. Furthermore, although no significant correlations were found between the small-world properties and clinical characteristics, a significant correlation was found between ISI changes and changes in functional connectivity between the right insula and left medial frontal gyrus (*r* = 0.45, *P* < 0.001). The correlation results are shown in Figure 5.

## Discussion

The functional networks of the three groups (PI at baseline, HCs, and PI at follow-up) all showed small-world topology. However, at baseline, the patients with PI showed impaired and inefficient small-world properties and aberrant functional segregation and functional integration compared with the HCs. Those properties showed a renormalization after individualized TMS treatment. Furthermore, decreased functional connectivity between the right insula and left medial frontal gyrus correlated with improvement in ISI scores. The current study highlights that insomnia is a disorder characterized by abnormal brain network connections, and that TMS may exert its treatment effects by reducing spontaneous hyper-connectivity in the default mode network and insula.

Multiple neuroimaging studies using fMRI have improved our understanding of the neural mechanisms underlying insomnia. Task-related fMRI studies have shown reduced activity in the medial frontal gyrus and inferior frontal gyrus in patients with PI during executive tasks (Altena et al., 2008; Drummond et al., 2013), and also abnormal activation to emotional stimuli within the amygdala (Baglioni et al., 2014). Resting state fMRI studies evaluating the amplitude of low-frequency fluctuations and regional homogeneity also found several abnormalities in patients with PI (Liu et al., 2016; Wang et al., 2016). Apart from regional impairments, functional connectivity, which measures the functionalcoordination between two regions, offers further information on the neural mechanisms of PI. Using seed-based region-to-region functional connectivity, Wang et al. (2017) found aberrant functional connectivity in the amygdala, insula, posterior cingulate gyrus, and hippocampus. However, many previous studies focused on impairments on circumscribed brain areas or functional connectivity, while the brain is a complex information processing system that coordinates multiple regions as a network, and it is therefore necessary to investigate the neural mechanism underlying PI using a network perspective.

Using graph theory-based analysis methods, we found that patients with PI exhibited poorly efficient small-world properties, and abnormal functional segregation (Cp and El) and functional integration metrics (Lp and Eg) at baseline. Functional segregation refers to local network efficiency, and reduced Cp and Eloc indicate that the “speed” of information transfer between adjacent nodes within a network is compromised (Duan et al., 2019). Functional integration ensures prompt interregional transfer of information, and abnormal enhanced functional integration (lower Lp and higher Eglob) indicates that the parallel information transfer in a brain network is impaired (Yu et al., 2017). Consistent with other observations, our study indicates that PI is a dysconnectivity disorder involving multiple neuronal circuits, rather than a focal pathology affecting a single region.

Hyperarousal processes play a key role in the pathophysiology of primary insomnia. Patients with PI are reported to show hyperarousal when awake, as well as during sleep (Lu et al., 2017). According to the hyperarousal theory, difficulty in falling and maintaining sleep is associated with greater functional connectivity. The reduced functional connectivity after TMS intervention suggests that TMS exerts its treatment effects by reducing hyper-connectivity. Impaired insular activity and abnormal connectivity have been noted in previous insomnia studies (Huang et al., 2012; Chen et al., 2014; Wang et al., 2016, 2017). The insula is an important brain region within the salience network, and plays an essential role in saliency detection; the reduced functional connectivity found in the right insula might indicate that its role in filtering out irrelevant sensory signals and transmitting salient information is compromised in patients with PI. Reduced functional connectivity was also found in the thalamus, which is a key area of the hyperarousal system, protecting sleep from external perturbations and selecting information to be projected to the cortex during normal sleep (Del Felice et al., 2012). The reduced functional connectivity found in the thalamus after TMS treatment may suggest an improvement in the power of the thalamus to block the transmission of sensory signals from the ascending reticular activating system and thalamus to the neocortex. We also found reduced functional connectivity in medial frontal gyrus, which is a core region of the default mode network (DMN). The DMN is active during rest and deactivates during goal directed behavior, and is involved in self-referential mental thought. The findings involving this network might be associated with rumination in PI patients, supporting this, a previous simultaneous TMS-EEG (electroencephalography) study found that after rTMS, information outflow was reduced in the frontal mid-line region but increased in the left temporal region (Song et al., 2019).

Several limitations to this study should be noted. First, the sample sizes were rather small, and a future study with a larger sample size is needed to allow generalization of the findings of this study. Second, we only explored the state of functional organization, and did not consider brain structural connectivity or other dynamic functional connectivity, which could provide more reliable information. Third, not all PI patients showed great improvement after SAINT administration, the underlying mechanism should be further studied in the future. Fourth, we did not control the placebo effect, further study should set up a placebo-controlled stimulations (Wu et al., 2020).

## Data Availability Statement

The raw data supporting the conclusions of this article will be made available by the authors, without undue reservation.

## Ethics Statement

Written informed consent was obtained from the individual(s) for the publication of any potentially identifiable images or data included in this article.

## Author Contributions

YZ, MX, and XM performed all data analysis and wrote the manuscript. KR, SQ, and Z-GH raised the conception of the study. XL and CS contributed to the collection of MRI data. SL and LL contributed to the manuscript revision. All authors read and approved the submitted version.

## Conflict of Interest

The authors declare that the research was conducted in the absence of any commercial or financial relationships that could be construed as a potential conflict of interest.

## Publisher’s Note

All claims expressed in this article are solely those of the authors and do not necessarily represent those of their affiliated organizations, or those of the publisher, the editors and the reviewers. Any product that may be evaluated in this article, or claim that may be made by its manufacturer, is not guaranteed or endorsed by the publisher.

## Footnotes

1
http://rfmri.org/dpabi

2
http://www.restfmri.net

3
www.fil.ion.ucl.ac.uk/spm

4
http://www.nitrc.org/projects/gretna/
